# Azadirachtin disrupts ecdysone signaling and alters sand fly immunity

**DOI:** 10.1186/s13071-024-06589-8

**Published:** 2024-12-20

**Authors:** Cecilia Stahl Vieira, Sara Bisogno, Marco Salvemini, Erich Loza Telleria, Petr Volf

**Affiliations:** 1 Department of Parasitology, Faculty of Science, Charles University, Prague, Czech Republic; 2 Department of Biology, University of Naples Federico II, Naples, Italy

**Keywords:** *Phlebotomus perniciosus*, Azadirachtin, Ecdysone, Antimicrobial peptides

## Abstract

**Background:**

Leishmaniasis is a group of neglected vector-borne diseases transmitted by phlebotomine sand flies. *Leishmania* parasites must overcome various defenses in the sand fly midgut, including the insects’s immune response. Insect immunity is regulated by the ecdysone hormone, which binds to its nuclear receptor (EcR) and activates the transcription of genes involved in insect immunity. However, the role of ecdysone in sand fly immunity has never been studied. *Phlebotomus perniciosus* is a natural vector of *Leishmania infantum*; here, we manipulated its neuroendocrine system using azadirachtin (Aza), a natural compound known to affect ecdysone synthesis.

**Methods:**

*Phlebotomus perniciosus* larvae and adult females were fed on food containing either Aza alone or Aza plus ecdysone, and the effects on mortality and ecdysis were evaluated. Genes related to ecdysone signaling and immunity were identified in *P. perniciosus*, and the expression of antimicrobial peptides (AMPs), *EcR*, the ecdysone-induced genes *Eip74EF* and *Eip75B*, and the transcription factor serpent were analyzed using quantitative polymerase chain reaction (PCR).

**Results:**

Aza treatment inhibited molting of first-instar (L1) larvae to L2, with only 10% of larvae molting compared to 95% in the control group. Serpent and *Eip74EF*, attacin, defensin 1, and defensin 2 genes were downregulated by Aza treatment in larvae. Similarly, Aza-treated adult females also presented suppression of ecdysone signaling-related genes and the AMPs attacin and defensin 2. Notably, all gene repression caused by Aza was reversed by adding ecdysone concomitantly with Aza to the larval or female food, indicating that these genes are effective markers for ecdysone repression.

**Conclusions:**

These results highlight the critical role of ecdysone in regulating the development and immunity of *P. perniciosus*, which potentially could interfere with *Leishmania* infection.

**Graphical Abstract:**

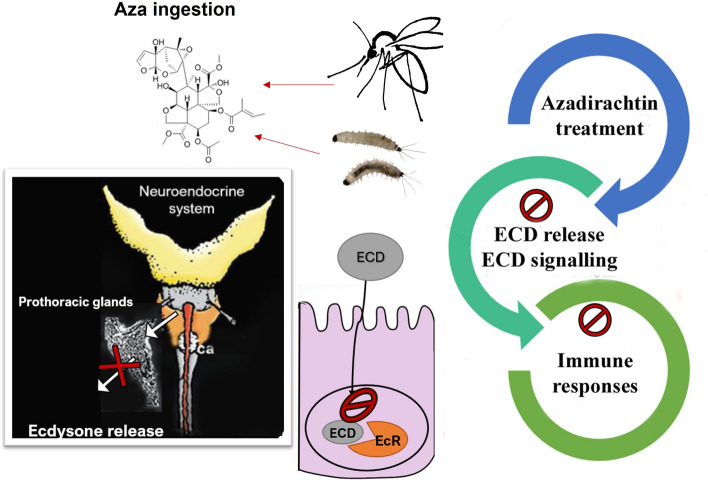

**Supplementary Information:**

The online version contains supplementary material available at 10.1186/s13071-024-06589-8.

## Background

Leishmaniasis encompasses a diverse group of neglected diseases caused by protists of the genus *Leishmania* (Kinetoplastida, Trypanosomatidae). These diseases rank among the most neglected globally, with approximately 350 million people at risk of infection. *Leishmania* parasites are transmitted to humans and animals by the bite of infected female sand flies (Diptera, Psychodidae, Phlebotominae). Within the sand fly gut, *Leishmania* undergoes a complex developmental process, including significant morphological and physiological changes; parasites must overcome various mechanical and physiological barriers plus the activation of the sand fly’s immune responses [1–3].

Signaling cascades such as the Toll and immune deficiency (Imd) pathways regulate the immune systems of insects. These cascades include Toll receptors or peptidoglycan-recognition proteins (PGRP), regulatory molecules such as cactus or caspar, transcription factors, and antimicrobial peptides (AMPs) as the main effector molecules [4]. In sand flies, the Toll and Imd pathways are activated upon infection by bacteria, yeast, and *Leishmania*, culminating with AMP expression [5–7].

AMPs are crucial in combating invading microbes and maintaining homeostasis of the microbiota in the insect gut [8–10]. For example, AMPs such as attacin and defensins have been shown to play a role in *Leishmania* infection, with their expression varying depending on the sand fly and the *Leishmania* species involved. Different defensins are upregulated in response to infections in different sand fly species, indicating a complex interaction between the parasite and the host’s immune response [7, 11]. This variability suggests that AMPs are part of the sand fly’s defense mechanism against pathogens, providing a dynamic barrier to parasite establishment and proliferation. However, the mechanisms by which the sand fly immune system interacts with *Leishmania* parasites are not completely understood.

Insect physiological processes, including immunity, are modulated and coordinated by the neuroendocrine system, which regulates the synthesis of hormones such as ecdysone. Ecdysone, a lipid-soluble steroid hormone, controls genes related to molting, metamorphosis, reproduction, and programmed cell death in insects [12–14]. Evidence indicates that the ecdysone signaling pathway also positively regulates humoral immunity in insects [15–19], initiating the expression of Imd signaling pathway-related genes through both peptidoglycan-recognition protein LC (PGRP-LC)-dependent and independent mechanisms [16, 20].

Although limited, studies on the role of the neuroendocrine system in vector immunity and parasite development inspired our group to explore this topic in sand flies. For example, in the triatomine *Rhodnius prolixus*, blocking ecdysone release by prothoracic glands was shown to suppress humoral immune responses such as lysozyme, AMPs, and phenoloxidase activity. This suppression impaired the clearance of systemic microbial infections, disrupted gut microbiota homeostasis, and increased mortality [19, 21, 22]. In the mosquito *Anopheles gambiae*, ecdysone signaling enhances immune responses and reduces *Plasmodium* infection, representing a potential target for interrupting parasite transmission [23–25]. In sand flies, the Imd pathway along with its transcription factor Relish is essential for regulating microbiota homeostasis and controlling *Leishmania* infection in the gut [26, 27]. However, the crosstalk between the neuroendocrine system and immune responses has yet to be explored in these insects.

Ecdysone is released by prothoracic glands and binds to the nuclear ecdysone receptor (EcR), which dimerizes with the ultraspiracle protein (USP) to form a functional nuclear receptor complex [28–30]. This complex binds to specific motifs near ecdysone-responsive target genes [31, 32]. This mechanism initiates the transcription of ecdysone-induced genes.

Early ecdysone-induced genes code for ecdysone-induced protein 74EF (*Eip74EF*), ecdysone-induced protein 75B (*Eip75B*), serpent, and others [16, 33, 34]. These proteins can inhibit their own transcription in a feedback loop while simultaneously initiating or enhancing the transcription of a broader array of late ecdysone-induced genes, establishing a hierarchical cascade of gene expression [35], which can include immune-related genes [16, 19]. To date, early ecdysone response genes, including *Eip74F*, *Eip75B*, and serpent, have not been studied in sand flies.

The intersection of the ecdysone signaling cascade and insect immunity presents an opportunity to manipulate hormonal signaling, influencing immune responses and ultimately affecting vector competence for pathogens [24, 36, 37]. Ecdysone regulates the expression of AMPs such as attacin, cecropin, and defensin, that are essential for the insect’s defense against microbial infections. However, the specific mechanisms underlying this regulation remain poorly understood.

To study the role of ecdysone signaling, one approach is to impair ecdysone production in insects through treatment with ecdysone antagonists such as azadirachtin (Aza), a triterpenoid compound extracted from the *Azadirachta indica* tree. Aza disrupts insect growth and molting by interfering with ecdysone production [21]. It primarily affects the insect neurosecretory system by inhibiting the release of morphogenetic peptide hormones like prothoracicotropic hormone, which regulates the prothoracic glands responsible for ecdysone secretion [38–40]. Furthermore, Aza can bind directly to the EcR, acting as an antagonist and disrupting the hormone’s function [41]. Consequently, insects treated with Aza experience significantly reduced ecdysone levels, leading to irregular molting, impaired growth, and higher mortality rates [42–44], confirming that Aza is a potent botanical insecticide.

Here, we were interested in investigating *Phlebotomus perniciosus*, the main vector of *Leishmania infantum*, a major etiological agent of visceral leishmaniasis (VL) in the Mediterranean region [45, 46]. Despite its epidemiological significance, the immunity and neuroendocrine regulation of *P. perniciosus* remain unknown. The present work focuses on unveiling genes related to ecdysone signaling in *P. perniciosus* and the crosstalk between the neuroendocrine system and immunity, using Aza as an ecdysone antagonist. By exploring the molecular mechanisms underlying ecdysone regulation, this study aims to identify potential targets for interfering with the sand fly immune system that could be further used in strategies to reduce vector competence.

## Methods

### *Phlebotomus perniciosus* colony maintenance

*Phlebotomus perniciosus* larvae and adult females were obtained from a colony originating from Murcia, Spain, maintained for two decades at the Department of Parasitology, Charles University, Prague, Czech Republic, under controlled conditions as described previously [47].

### Aza and ecdysone treatment in *P. perniciosus* larvae

*Phlebotomus perniciosus* larvae were fed a diet containing composted and powdered rabbit feces, as described previously [47]. Aza (Sigma-Aldrich, Darmstadt, Germany) and α-ecdysone (Sigma-Aldrich) (the precursor of 20E) were first dissolved in 250 μl of 99% ethanol P.A. and then diluted in 750 μl of 0.9% NaCl (1:4 ethanol/saline), resulting in final concentrations of 1 and 2.5 μg/ml, respectively. Different volumes of diluted Aza were initially tested to determine the appropriate method for incorporating the liquid Aza into the powdered sieved larval food suitable for first-instar larvae (L1).

To standardize the volume of Aza diluent that could be mixed into the L1 food, we aimed for a pasty texture that would allow the larvae to feed and develop normally. The chosen mixture consisted of 35 mg of larval food plus 125 μl of 0.9% NaCl. Consequently, Aza and ecdysone final concentrations were 1 μg and 2 μg per milligram of L1 food, respectively. These concentrations were based on previous studies with *Lutzomyia longipalpis* [42]. This mixture was sufficient to feed approximately 200 L1 larvae in a small plaster pot for 2 days.

The experimental groups were as follows: (a) control group: L1 food plus 0.9% NaCl; (b) Aza group: L1 food plus 1 μg/mg of Aza; (c) Aza + Ecd group: L1 food containing 1 μg/mg of Aza plus 2 μg/mg of ecdysone; (d) Ecd group: L1 food containing 2 μg/mg of ecdysone. Each group consisted of three pots, each containing approximately 200 larvae. Each pot received L1 food equivalent to 35 mg of the dry powder mixture containing the specified chemicals, provided twice until the first molting to L2 larvae was observed in the control group (around 15 days after larval hatching week from eggs).

Larval development was monitored daily, considering the size of larvae and the number of caudal setae (two in L1 and four in L2). Adult emergence was observed following standard colony maintenance. The larvae were collected in pools of 15 individuals from each group at 1 and 7 days after first feeding (DAF) for subsequent gene expression analysis. The larval collection was random, without distinction of larval stage, ensuring that the results reflected the overall impact of the treatments on the larval populations. The experiments were conducted in triplicate.

### Aza and ecdysone treatment in *P. perniciosus* females

Four hundred recently emerged *P. perniciosus* females were randomly selected, separated into four cages, and maintained without sugar for 24 h. Then each cage received a small petri dish with a drop (20 µl) of sterile filtered sucrose solution containing the following: (a) control group; 0.9% NaCl; (b) Aza group; 1 μg/mg of Aza; (c) Aza + Ecd group; 1 μg/mg of Aza plus 2 μg/mg of ecdysone; (d) Ecd group: 2 μg/mg of ecdysone. Brilliant blue food dye was added to the sucrose solution to facilitate the differentiation between sugar-fed and non-fed females. Non-fed females were discarded from the experiment. At 1 and 3 DAF, two pools of 10 insects from each group were collected, placed in 1.5 ml tubes, and kept in an −80 º freezer before RNA extraction. The mortality of females was followed daily for 3 DAF.

### RNA extraction and cDNA synthesis

Total RNA was extracted from whole-body samples of *P. perniciosus* larvae and females using the High Pure RNA Isolation Kit (Roche, Pleasanton, CA, USA), following the manufacturer’s instructions. Synthesis of complementary DNA (cDNA) was carried out on the same day using the Transcriptor First Strand cDNA Synthesis Kit (Roche) using up to 1 μg of total RNA and anchored-oligo dT_(18)_ primers in a 20 μl reaction following the manufacturer’s instructions. The cDNA concentration was quantified using the Quantus fluorometer (Promega, Madison, WI, USA).

### Identification of genes related to the ecdysone signaling pathway and AMPs in *P. perniciosus*, primer design and primer validation

The genes coding the AMPs attacin (*att*), defensin 1 (*def 1*), and defensin 2 (*def 2*), the ecdysone receptor (*EcR*), and the early ecdysone response genes *Eip74EF*, *Eip75B*, and serpent were searched by performing a TBLASTN search against the available *P. perniciosus* adult transcriptome database [48] using the following *Phlebotomus papatasi* protein sequences: *att*: PPAI003791; *def 1*: PPAI004256; *def 2*: PPAI010650; and the following *Drosophila melanogaster* protein sequences: EcR: FBpp0291631; *Eip74EF*: FBpp0074967; *Eip75B*: FBgn0000568; serpent: FBpp0082669; as virtual probes. The transcript sequences are available in Supplementary file 1.

The primers were designed using Geneious Prime^®^ 2024.0.5 software (Biomatters, Auckland, New Zealand) under the following conditions: melting temperatures between 59.0 and 61.0 °C, GC content between 40 and 60%, nucleotide length between 18 and 24, and amplicon length of between 150 and 225 bases. The primers are listed in Table 1.

**Table 1:** List of primers designed and used for qPCR reactions

Gene	Primer name	Primer sequence
*GAPDH*	PperGAPDH—F	5′-AGTCCACGGGAGTGTTTACCA-3′
	PperGAPDH—R	5′-CGCCGCCCTTGAGATG-3′
*GPDH*	PperGPDH—F	5′-TTCCAAAGACGCCGACATC-3′
	PperGPDH—R	5′-GGCCAAGCCACGAATGAA-3′
Ecdysone receptor	PperEcR—F	5′-TGTCAAAGTAGAGCCAATACACG-3′
	PperEcR—R	5′-GATGACAACCAATCGTCACCTTC-3′
Ecdysone-induced protein 74EF	PperEip74EF—F	5′-CCGATGAGATTCTCCGCCAA-3′
	PperEip74EF—R	5′-CGGGCAGGTCATTGTTGTTC-3′
Ecdysone-induced protein 75B	PperEip75B—F	5′-ATGTGGTCGATGGAGGAGGA-3′
	PperEip75B—R	5′-CTGGAGACTGAGCCCATTGG-3′
Serpent	PperSRP—F	5′-CTCGGCGAACCAGTGTGTAA-3′
	PperSRP—R	5′-TGGCTTCCTTTTGCGAGTCT-3′
Attacin	PperAtt—F	5′-CGACAGGACCCACAAGCATT-3′
	PperAtt—R	5′-GTCCTTCGGCAGTCAGACTC-3′
Defensin 1	PperDef1—F	5′-TGTGAGTTGTGACGTCCTGG-3′
	PperDef1—R	5′-ACTTAACGACGACACCTGCAT-3′
Defensin 2	PperDef2—F	5′-TTGTTGGAGTCGTCAGTGCT-3′
	PperDef2—R	5′-CCAAATAGGGCTTCCGGGAA-3′

Conventional polymerase chain reaction (PCR) was performed for each target gene studied here using GAPDH as a positive control to confirm the targeted genes. Reactions were performed using the EmeraldAmp GT PCR Master Mix (TaKaRa, Shiga, Japan) according to the manufacturer’s instructions and visualized in a 2% agarose gel. Cycling conditions were as follows: 95 °C for 3 min; 34 amplification cycles (95 °C for 30 s; 60 °C for 30 s, 72 °C for 1 min); and 72 °C for 5 min. Amplicons were visualized in a 1% agarose gel to confirm the product size. The products were also excised from gels and purified using the Agarose Gel DNA Extraction Kit** (**Roche). The purified DNA samples were sequenced for identity confirmation.

A standard curve was performed for each target gene using a serial dilution of *P. perniciosus* cDNA as a template to assess the primer efficiencies on the qPCR assays [49]. Primers presenting efficiency between 90 and 110% and showing a single peak in the derivative melting curve were chosen [50, 51].

### *Phlebotomus perniciosus* gene expression

The expression of AMPs *att*, *def 1*, *def 2*, and the ecdysone-related genes *EcR*, *Eip74EF*, and *Eip75B* and serpent was evaluated through quantitative PCR. Quantitative PCR (qPCR) was prepared with 1 ng of cDNA samples, gene-specific primers, and SYBR Green PCR Master mix (Roche) in a 384-well plate to detect the expression of target genes using a LightCycler 480 thermocycler (Roche) in a final volume of 10 μl. Relative gene expression was calculated relative to *P. perniciosus* endogenous control genes *GAPDH* and *GPDH* [48] using the following cycling conditions: 95 °C for 10 min enzyme activation, initial denaturation at 95 ºC for 10 s, annealing at 60 °C for 20 s; extension at 72 °C for 45 s, repeated 45 times. Expression levels were expressed as the fold change compared to the control groups, using the Pfaffl method [52]. The three independent experiments were performed with two pools of each sample in technical duplicates (*n* = 6).

### Statistical analysis

The experimental data were analyzed using GraphPad Prism 10 software (GraphPad Software, CA, USA). A Student *t*-test was used to evaluate significant differences in gene expression levels between two groups at each time point and to assess differences in mortality rates between experimental and control groups at each time point separately. The significant differences observed among groups are detailed in the respective figures and figure legends. A threshold of *P* < 0.05 was used to determine statistical significance.

## Results

### Development of *P. perniciosus* larvae after hormonal modulation

*Phlebotomus perniciosus* L1 larvae were fed L1 food containing Aza, Aza plus ecdysone, or ecdysone, to observe the effect of these components on the molting process from L1 to L2.

**Fig. 1:**
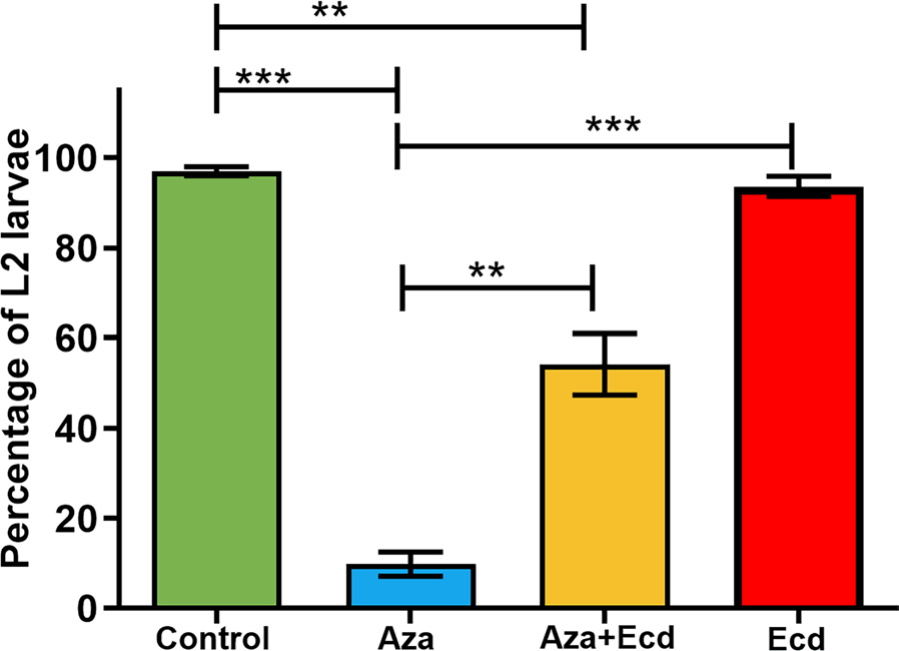
Effects of Aza and ecdysone treatment on the molting of *P. perniciosus* larvae. The larvae were fed with food containing 0.9% NaCl (control, green column), Aza (1 μg/mg, blue column), Aza (1 μg/mg) combined with ecdysone (2 μg/mg, yellow column), and ecdysone alone (2 μg/mg, red column). L1 molting to L2 was monitored up to 15 days after eclosion. Bars represent the mean ± standard error of the mean (SEM) of three independent experiments (*n* = 15). Means were compared using Student’s *t*-test: *P < 0.05, **P < 0.01, ***P < 0.001

The group fed with Aza showed significant inhibition of ecdysis when compared to the control group (Fig. 1, *P* < 0.001). This effect of Aza on molting was partially rescued by the simultaneous addition of ecdysone in the L1 food (Fig. 1, *P* < 0.01). The proportion of L2 in the control group was similar to the ecdysone group but significantly higher than in the Aza and Aza plus ecdysone groups (Fig. 1, *P* < 0.01, Fig. 1, *P* < 0.05).

### Sand fly larvae: hormonal modulation affects the expression of genes related to ecdysone signaling

The effects of hormonal modulation on the expression of genes associated with ecdysone signaling in *P. perniciosus* larvae were studied on days 1 and 7 after feeding.

**Fig. 2:**
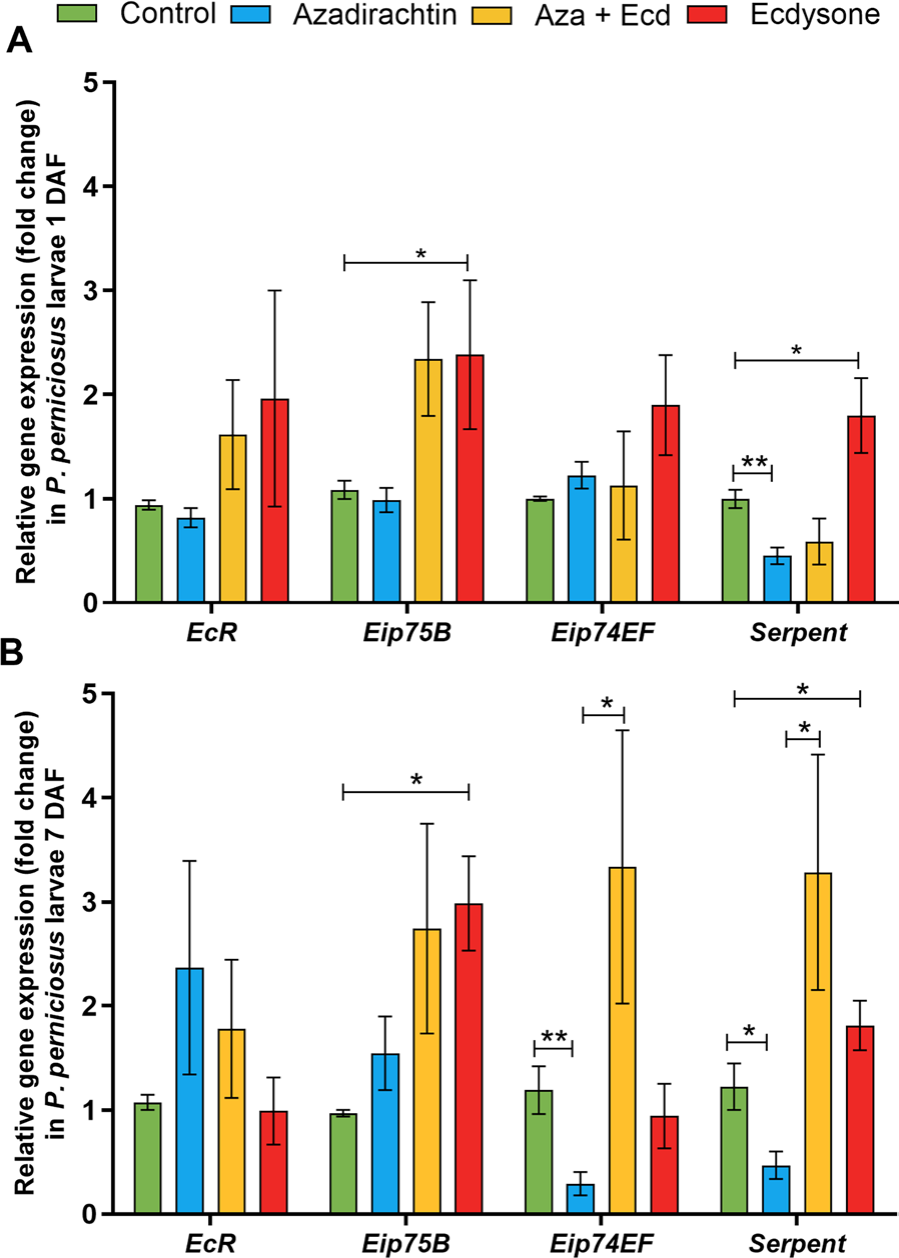
Effects of Aza and ecdysone on the expression of ecdysone signaling pathway-related genes in *P. perniciosus* larvae. *Phlebotomus perniciosus* larvae were previously fed with food containing 0.9% NaCl, Aza [1 μg/mg], Aza [1 μg/mg] plus ecdysone [2 μg/mg], and ecdysone [2 μg/mg]. The gene expression of *EcR*, *Eip75B*, *Eip74EF*, and serpent was evaluated **A** 1 day and **B** 7 days after feeding. Bars represent the mean ± standard error of the mean (SEM) of three independent experiments (*n* = 15). Means were compared using Student’s *t*-test; *P < 0.05, **P < 0.01

On day 1, Aza treatment did not alter the expression of *EcR*, *Eip75B*, and *Eip74EF* (Fig. 2A) but significantly decreased the expression of *Serpent* (*P* < 0.01, Fig. 2A) in *P. perniciosus* L1. Ecdysone did not counteract Aza suppression of *Serpent* (Aza + ecdysone group), but in larvae fed on ecdysone, *Eip74EF* and *Serpent* expression was higher than in control insects (*P* < 0.05, Fig. 2A).

On day 7, Aza treatment significantly downregulated *Eip74EF* and serpent (*P* < 0.01; *P* < 0.05), while *EcR* and *Eip75B* expression remained unaltered (Fig. 2B). The suppression of *Eip74EF* and serpent by Aza treatment was counteracted by ecdysone (Aza + Ecd group) (*P* < 0.05, Fig. 2B). However, ecdysone supplementation (Ecd group) only upregulated the expression of *Eip75B* and serpent (*P* < 0.05, Fig. 2B).

### Sand fly larvae: hormonal modulation affects the expression of AMP genes

**Fig. 3:**
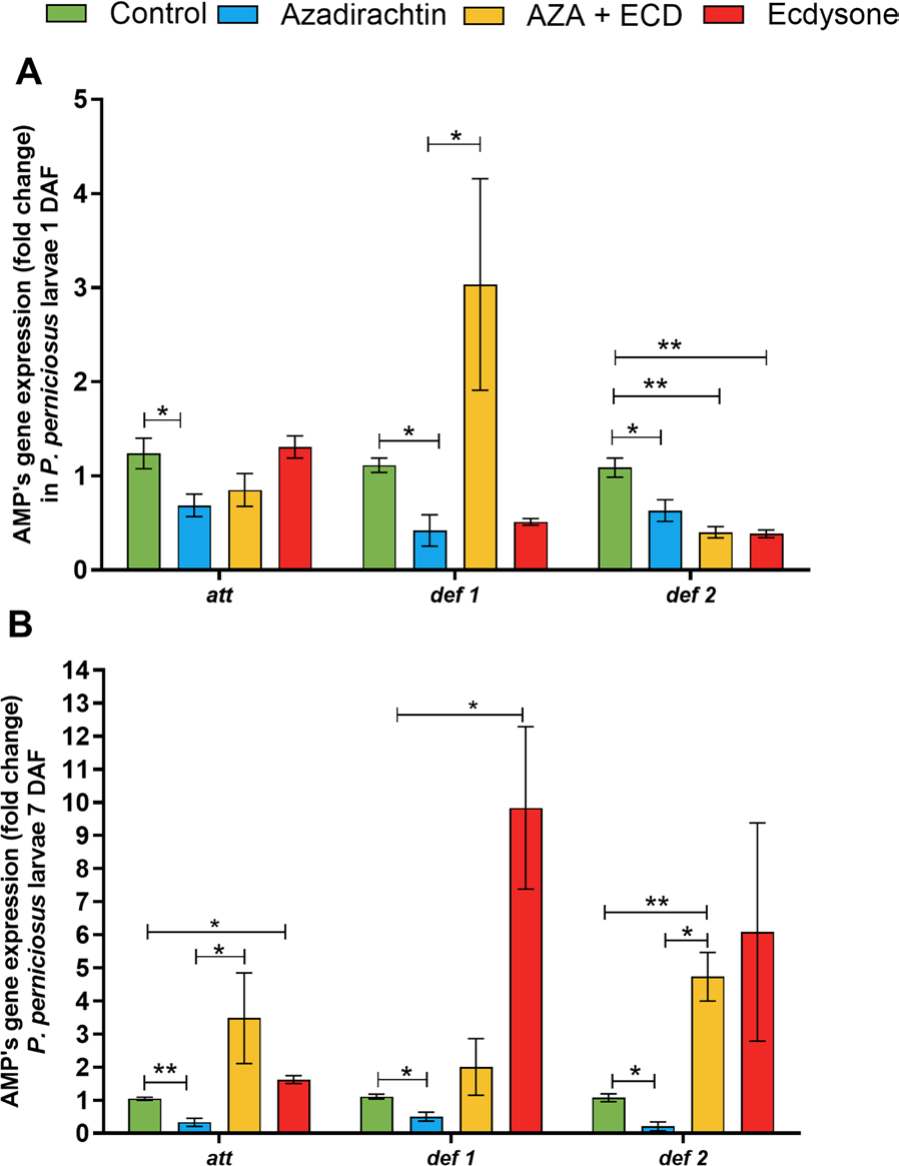
Effects of Aza and ecdysone treatment on the expression of antimicrobial peptides in *P. perniciosus* larvae. *Phlebotomus perniciosus* larvae were previously fed with food containing 0.9% NaCl, Aza [1 μg/mg], Aza [1 μg/mg] plus ecdysone [2 μg/mg], and ecdysone [2 μg/mg]. The gene expression of attacin (*att*), defensin 1 (*def 1*), and defensin 2 (*def 2*) was evaluated **A** 1 day and **B** 7 days after feeding. Bars represent the mean ± standard error of the mean (SEM) of three independent experiments (*n* = 15). Means were compared using Student’s *t*-test; *P < 0.05, **P < 0.01

The gene expression of AMPs *att*, *def 1*, and *def 2* was analyzed on days 1 and 7 after hormonal modulation in *P. perniciosus* larvae. On day 1, *att*, *def 1*, and *def 2* gene expression was significantly reduced in larvae fed on Aza (*P* < 0.05, Fig. 3A). The reduction in *def 1* expression was rescued by the concomitant addition of ecdysone if compared to the control group (group Aza + Ecd), while it was increased if compared to the Aza group (*P* < 0.05, Fig. 3A). On the other hand, ecdysone supplementation (Ecd group) did not induce AMP expression (Fig. 3A).

On day 7, the expression of all AMPs remained significantly reduced in the Aza group (*P* < 0.001; *P* < 0.05, Fig. 3B). Furthermore, ecdysone not only increased the expression of *att* and *def 2*, as observed in the Aza + Ecd treatment group compared to Aza treatment group (*P* < 0.05, Fig. 3B), but also increased their expression compared to the control group (*P* < 0.01, Fig. 3B). Surprisingly, *def 1* expression was increased tenfold by ecdysone supplementation (Ecd group) (*P* < 0.05, Fig. 3B), whereas no other AMP was upregulated by hormonal addition to *P. perniciosus* larvae.

### Sand fly females: hormonal modulation affects the expression of genes related to ecdysone signaling

**Fig. 4:**
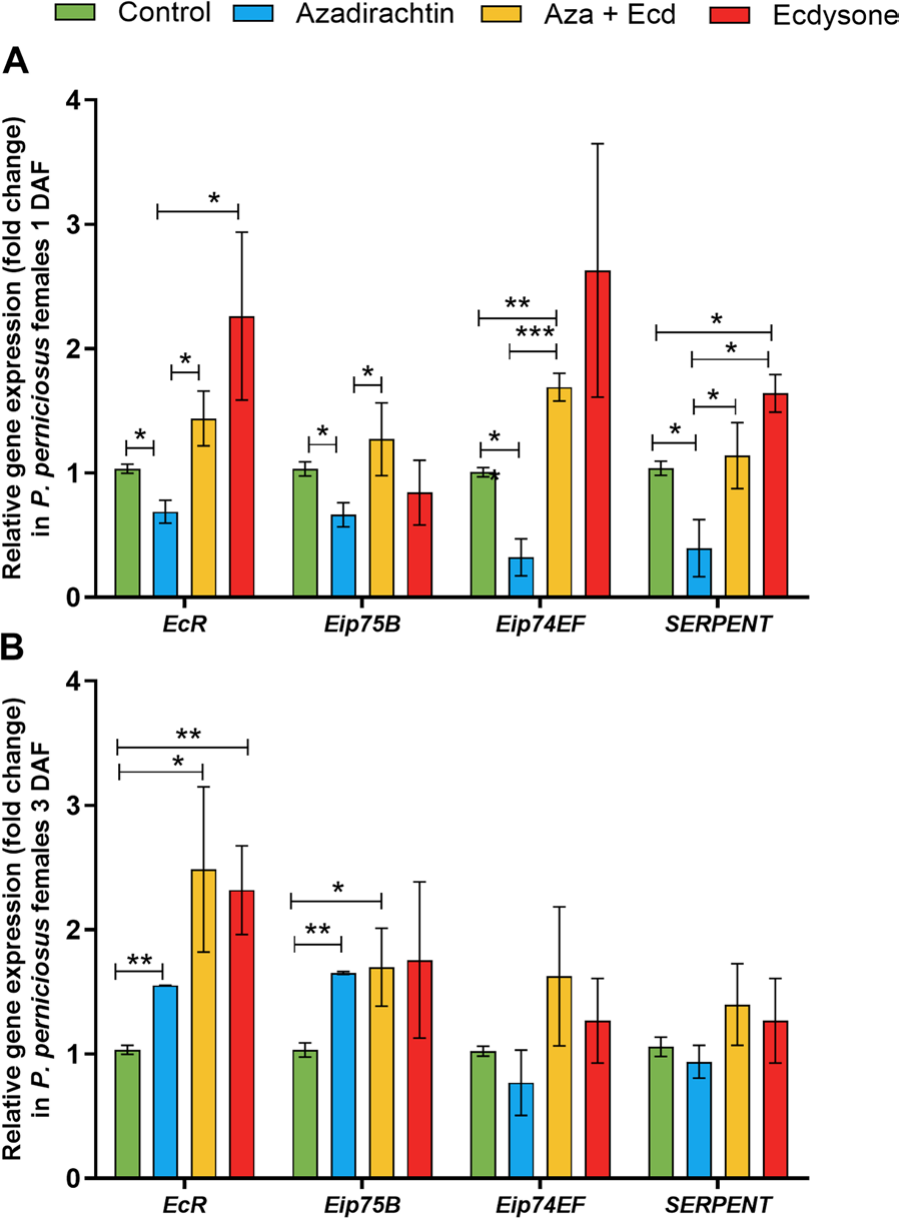
Effects of Aza and ecdysone treatment on the expression of ecdysone signaling pathway-related genes in *P. perniciosus* females. Females were previously fed on sucrose solution containing 0.9% NaCl, Aza [1 μg/mg], Aza [1 μg/mg] plus ecdysone [2 μg/mg], and ecdysone [2 μg/mg]. The gene expression of *EcR*, *Eip75B*, *Eip74EF*, and serpent was evaluated **A** 1 day and **B** 7 days after feeding. Bars represent the mean ± standard error of the mean (SEM) of three independent experiments (*n* = 15). Means were compared using Student’s *t*-test; *P < 0.05, **P < 0.01, ***P < 0.001

Aza and ecdysone treatment did not alter mortality rates in *P. perniciosus* females (Additional file 1) but affected the gene expression of ecdysone-induced proteins and *EcR* in *P. perniciosus* females. On day 1, *EcR*, *Eip75B*, *Eip74F*, and *Serpent* expression were significantly reduced in the Aza-treated group (*P* < 0.05, Fig. 4A). Ecdysone reversed the inhibitory effects of Aza on all ecdysone-related genes (Aza + Ecd group) (*P* < 0.05; *P* < 0.001, Fig. 4A). The supplementation of ecdysone (Ecd group) significantly upregulated serpent expression at 1DAF (*P* < 0.05, Fig. 4A).

On day 7, *EcR* and *Eip75B* expression was significantly increased in females fed on Aza (*P* < 0.01, Fig. 4B) as well in the Aza + Ecd group (*P* < 0.05, Fig. 4B). Finally, in the group fed ecdysone, a significant increase in *EcR* expression was observed (*P* < 0.01, Fig. 4B).

### Sand fly females: hormonal modulation affects the expression of AMP genes

**Fig. 5:**
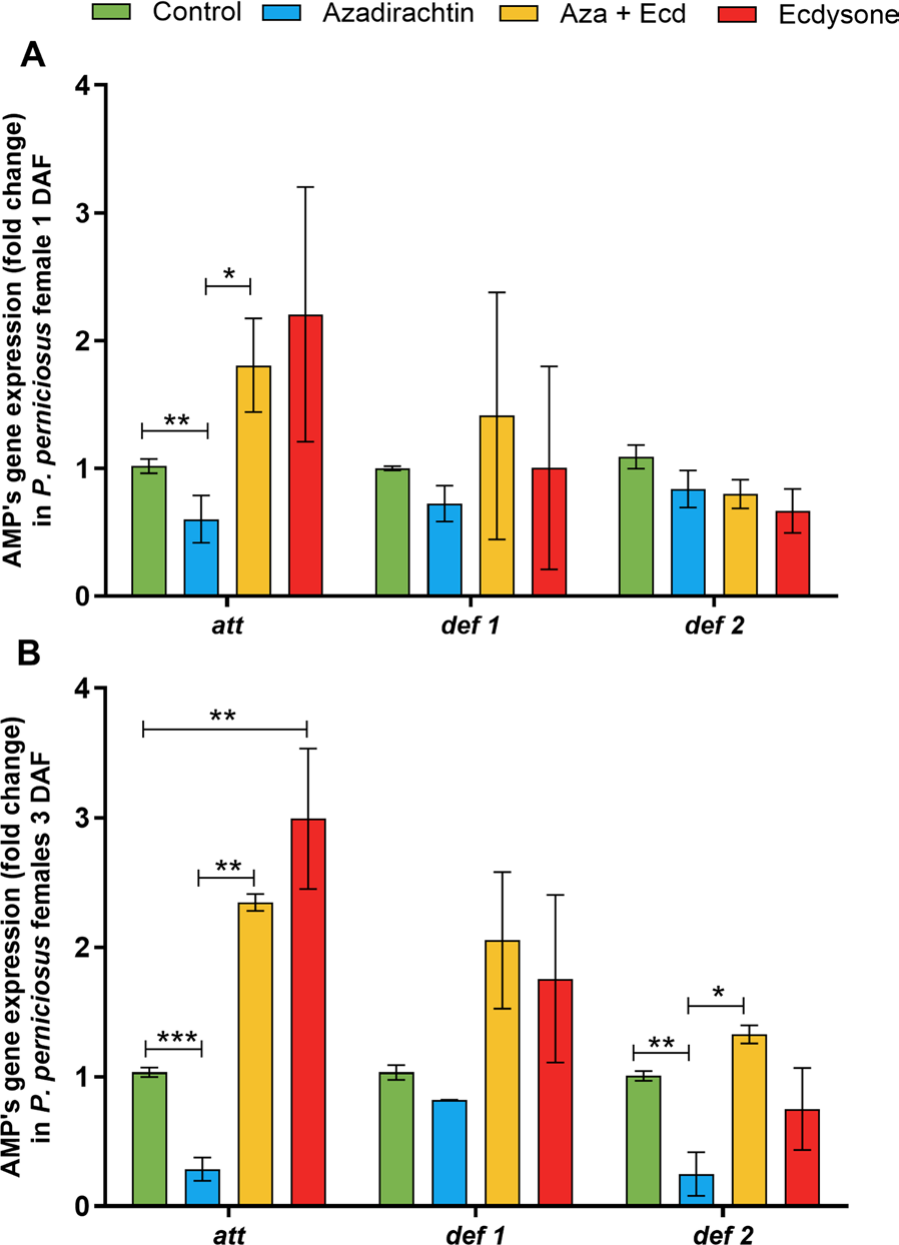
Effects of Aza and ecdysone treatment on the expression of antimicrobial peptides in *P. perniciosus* females. Females were previously fed on sucrose solution containing 0.9% NaCl, Aza [1 μg/mg], Aza [1 μg/mg] plus ecdysone [2 μg/mg], and ecdysone [2 μg/mg]. The gene expression of attacin (*att*), defensin 1 (*def 1*), and defensin 2 (*def 2*) was evaluated 1 day (**A**) and **B** 7 days after feeding. Bars represent the mean ± standard error of the mean (SEM) of three independent experiments (*n* = 15). Means were compared using Student’s *t*-test; *P < 0.05, **P < 0.01

On day 1, Aza treatment significantly reduced *att* expression (*P* < 0.01, Fig. 5A). The ecdysone counteracted Aza inhibitory effects on *att* expression, as observed in the Aza + Ecd treatment group compared to the Aza treatment group (*P* < 0.05, Fig. 5A).

On day 7, *att* expression remained significantly lower in the Aza-treated group (*P* < 0.001, Fig. 5B). Additionally, *def 2* expression was also downregulated by Aza treatment (*P* < 0.01, Fig. 5B). Ecdysone counteracted the inhibitory effects of Aza, as observed in the Aza + Ecd-treated group compared to the Aza-treated group (*P* < 0.01; *P* < 0.05, Fig. 5B). Finally, the ecdysone-fed group presented increased expression of *att* (*P* < 0.01, Fig. 5B).

## Discussion

### Aza altered larvae development by inhibiting *Eip74EF* and *Serpent* expression

Aza, derived from the neem tree (*Azadirachta indica*), is a potent plant insecticide known for interfering with insect growth and development [40, 53]. The mechanisms of action of Aza include inhibition of ecdysone synthesis by the prothoracic gland and release in the hemolymph, leading to reduced levels of this crucial hormone and subsequent effects on gene expression in insects [54–56]. In *D. melanogaster*, Aza inhibits the ecdysone 20-monooxygenase enzyme [55]. It affects hemolymph ecdysteroid levels by suppressing the release of morphogenetic peptide hormones [53], which are necessary for ecdysteroid production and vital for normal larval development and metamorphosis, respectively.

In agreement, our results demonstrated that Aza oral treatment significantly inhibited the larvae molting process from L1 to L2, highlighting its interference with ecdysone levels and ecdysis, as already described in other dipterans [18, 57, 58], including the sand fly *L. longipalpis* [42]. Ecdysone partially mitigated this effect, though the proportion of larvae reaching L2 was still lower than controls. Notably, no adults emerged in the Aza-treated groups, indicating profound developmental disruption. Earlier studies in *D. melanogaster*, *Aedes aegypti*, *Manduca sexta*, and *R. prolixus* demonstrated that Aza treatment often causes a delay or permanent blockage of molting due to decreased ecdysteroid levels [59, 60]. These results confirm that Aza is an effective tool for studying ecdysone signaling and its downstream effects.

Here, Aza oral treatment generally suppressed the expression of early ecdysone-induced genes, but not *EcR*, in *P. perniciosus* larvae, though the timing and duration of this modulation varied between the two developmental stages. It was reported that Aza treatment impaired insect growth and development by downregulating *EcR* gene expression and other ecdysone-induced genes in the Japanese pine sawyer beetle *Monochamus alternatus* [61], *Drosophila* [54], and *Spodoptera frugiperda* [62]. The lack of *EcR* modulation in *P. perniciosus* larvae following Aza treatment suggests a species-specific resistance mechanism, potentially involving alternative hormonal pathways.

Temporal analysis of early ecdysone-induced genes in *P. perniciosus* larvae revealed that only the serpent gene was immediately and persistently suppressed after Aza treatment, while *Eip74EF* repression was not immediate, only occurring 7 DAF. Ecdysone concomitant supplementation with Aza partially counteracted *Eip74EF* and serpent suppression. Although we cannot detect a diminishing *EcR* in larvae after Aza treatment, the receptor function could be altered, as previously suggested. It was observed that while Aza significantly affected other genes, there was no significant change in the expression of *EcR*, although diminished expression was observed in its related gene *USP*, a heterodimer of the ecdysone receptor, which forms a functional nuclear receptor complex [61]. In *Bombyx mori*, it was shown that Aza did not directly alter gene *EcR* expression but influenced intracellular calcium release, suggesting that Aza may indirectly alter *EcR* function via calcium signaling pathways rather than by directly modulating gene expression [56]. Alterations in the EcR function could explain the effects on the expression of *Eip74EF* and serpent in *P. pernicisosus* larvae.

*Eip74EF* and serpent are transcription factors that activate several downstream genes in the ecdysone signaling cascade. The *Eip74EF* gene plays a crucial role during the ecdysone signaling pathway, acting as a transcription factor regulating the expression of developmental genes during insect molting [63, 64]. Studies in *Drosophila* and other insects have highlighted *Eip74EF*’s function in cyst differentiation, cell death, and tissue morphogenesis [65]. Comparative studies confirm the conserved nature of *Eip74EF*’s role across different species [66]. Serpent is a GATA transcription factor that is crucial during embryo fat body and hemocyte differentiation [67, 68] and is also necessary for activating gene expression in the mature fat body during larval development [69]. In this sense, the *Eip74EF* and serpent gene suppression could be related to the molting impairment observed in *P. perniciosus* larvae fed on Aza and seems to be a crucial gene for larvae development until adults.

### Aza altered adult female expression of ecdysone signaling-related genes

In *P. perniciosus* females, ingestion of Aza led to significant suppression of *EcR* expression at 1 DAF, which correlated with the downregulation of *Eip74EF*, *Eip75B*, and serpent. This effect was not sustained by 7 DAF. Interestingly, Aza did not alter *EcR* expression in larvae. Nevertheless, in adult females, the *EcR* suppression suggests a differential response to ecdysone inhibition between developmental stages, possibly due to varying sensitivity to Aza or distinct hormonal compensatory mechanisms at different life stages.

Exogenous ecdysone supplementation is known to increase ecdysone titers in insects [70, 71] and to upregulate *EcR* expression [72, 73]. In line with this, we observed upregulation of *EcR* in response to ecdysone supplementation in *P. perniciosus* females, which could be connected to increased ecdysone levels in adult sand flies. Conversely, insects treated with Aza exhibited reduced ecdysone levels [60, 70]. The reduced ecdysone levels we observed in AZA-treated *P. perniciosus* could be linked to the downregulation of *EcR* expression or diminished EcR/USP complex activity. Taken together, *EcR* impairment directly influences the transcription of *Eip74EF*, *Eip75B*, and *Serpent*.

### Aza induced immunosuppression in larvae and adult females by altering ecdysone signaling: discussing the roles of *Eip74EF*, *Eip75B*, and *Serpent* in *P. perniciosus* immunity

We used Aza in *P. perniciosus* larvae and females to disrupt ecdysone signaling and assess alteration of insect immune effectors. Since ecdysone is critical for both development and immune regulation [16, 19], its disruption by Aza affects both processes. In agreement, AMPs have cis-regulatory elements for EcR-USP receptor complex binding, indicating that AMP regulation is ecdysone-dependent [16]. Here, the recovery of AMP expression upon treatment with exogenous ecdysone indicates that ecdysone counteracts Aza’s immunosuppressive effects.

Although the role of the *Eip74EF* gene in insect immunity is not well documented, emerging evidence suggests it may contribute to immune responses. For instance, knocking down *Eip74EF* in adult *Drosophila* reduces the expression of the Imd pathway receptor PGRP-LC and the AMPs cecropin and diptericin, thereby compromising the flies’ resistance to bacterial infections, resulting in increased mortality rates [16, 74]. Our results indicate that *Eip74EF* could be connected with attacin and defensin expression in *P. perniciosus*, thereby establishing a connection between hormonal regulation and immune defense mechanisms in sand flies.

The ecdysone signaling pathway includes feedback mechanisms where early response genes like *Eip75B*, which acts as a transcriptional repressor, inhibit their own expression and that of other early genes to fine-tune the hormonal response [16, 29, 75, 76]. *Eip75B* has been implicated in regulating *Drosophila* immunity by interfering with the expression of AMPs. The Eip75B protein may directly bind to AMP gene promoter regions to repress transcription and compete with nuclear factor kappa beta (NF-κB)-like transcription factors for binding sites, affecting AMP expression [16, 77, 78]. While the precise role of *Eip75B* in *P. perniciosus* biology remains unclear, we propose that *Eip75B* could modulate AMPs, altering basal immunity and disturbing gut homeostasis when ecdysone signaling is compromised.

It has been shown that serpent activates AMP expression by binding to GATA motifs in the regulatory regions of these genes, which are often located near REL sites [16, 69, 79–81]. The synergy between REL and GATA transcription factors is a common feature in regulating immune genes, as demonstrated in *Drosophila*, where serpent and REL-containing transcription factors such as Dorsal and Dif collaborate to ensure robust immune responses during infection [16, 82]. Moreover, a study on *Anopheles aquasalis* demonstrated that serpent is essential for immunity against both *Plasmodium* and bacterial infections [83], further supporting the conserved role of serpent in insect vector immunity. In our study, Aza-induced ecdysone inhibition suppressed serpent expression, leading to reduced AMP levels in *P. perniciosus* larvae and females. These effects were reversed by exogenous ecdysone supplementation. While our study did not specifically test insect susceptibility to infection, we hypothesize that suppressing immune-related genes, particularly during molting, may affect the insect’s overall immune competence.

It has been demonstrated that the Imd pathway regulates attacin and defensins in sand flies [6, 84], and impairment of the Imd pathway increases the sand fly’s susceptibility to *Leishmania* infection [27]. Taken together, our results highlight the pivotal role of serpent in regulating attacin and defensins and illustrate how ecdysone signaling can modulate this crosstalk, ultimately affecting the immune competence of *P. perniciosus*. We plan to explore this further in our next manuscript, focusing on the effects of ecdysone impairment in *P. perniciosus* immunity in the context of *L. infantum* infection.

## Conclusions

This study demonstrates that Aza disrupts ecdysone signaling, significantly affecting both developmental and immune processes in *P. perniciosus*. The downregulation of key ecdysone-induced genes and AMPs in both larval and adult stages highlights the crucial role of ecdysone signaling in immune regulation, which is essential for maintaining immune competence throughout development. Moreover, the partial restoration of gene expression via exogenous ecdysone supplementation indicates that neuroendocrine modulation can potentially influence insect immunity. These findings enhance our understanding of the interplay between neuroendocrine and immune systems in *P. perniciosus* biology and suggest new avenues for developing novel vector control strategies targeting hormone-regulated pathways.

## Supplementary Information

Additional file 1.

Supplementary file 1.

## Data Availability

All data generated and analysed during this study are included in this published article (and in Supplementary file 1).
