# Supplementary material for: Azadirachtin disrupts ecdysone signaling and alters sand fly immunity
Source: Parasit Vectors. 2024 Dec 20;17:526. doi: 10.1186/s13071-024-06589-8 (PMC11662615; doi:10.1186/s13071-024-06589-8)
Supplement: Supplementary file 1 — Additional file 1. [file 13071_2024_6589_MOESM1_ESM.docx]

Azadirachtin disrupts ecdysone signaling and alters sand fly immunity

Additional files


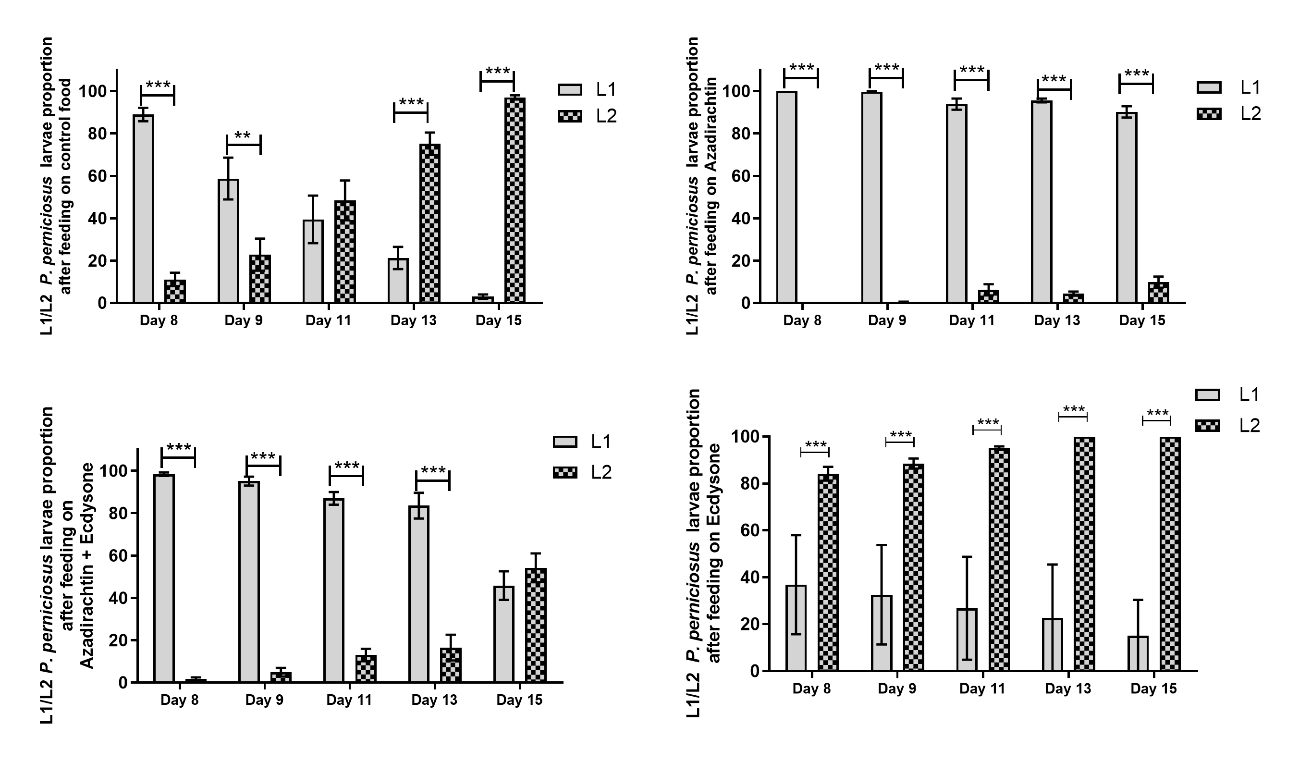


**Additional file 1: Effects of azadirachtin and ecdysone treatment on the molting of *P. perniciosus* larvae. The larvae were fed with food containing: 0.9** % **NaCl (control, green column), azadirachtin (1 μg/mg, blue column), azadirachtin (1 μg/mg) combined with ecdysone (2 μg/mg, yellow column), and ecdysone alone (2 μg/mg, red column). L1 molting to L2 was monitored up to 15 days after eclosion. Bars represent the mean ± SEM of three independent experiments (n = 15). Means were compared using Student’s T-test: **p *<* 0.01, ***p<0.001.**


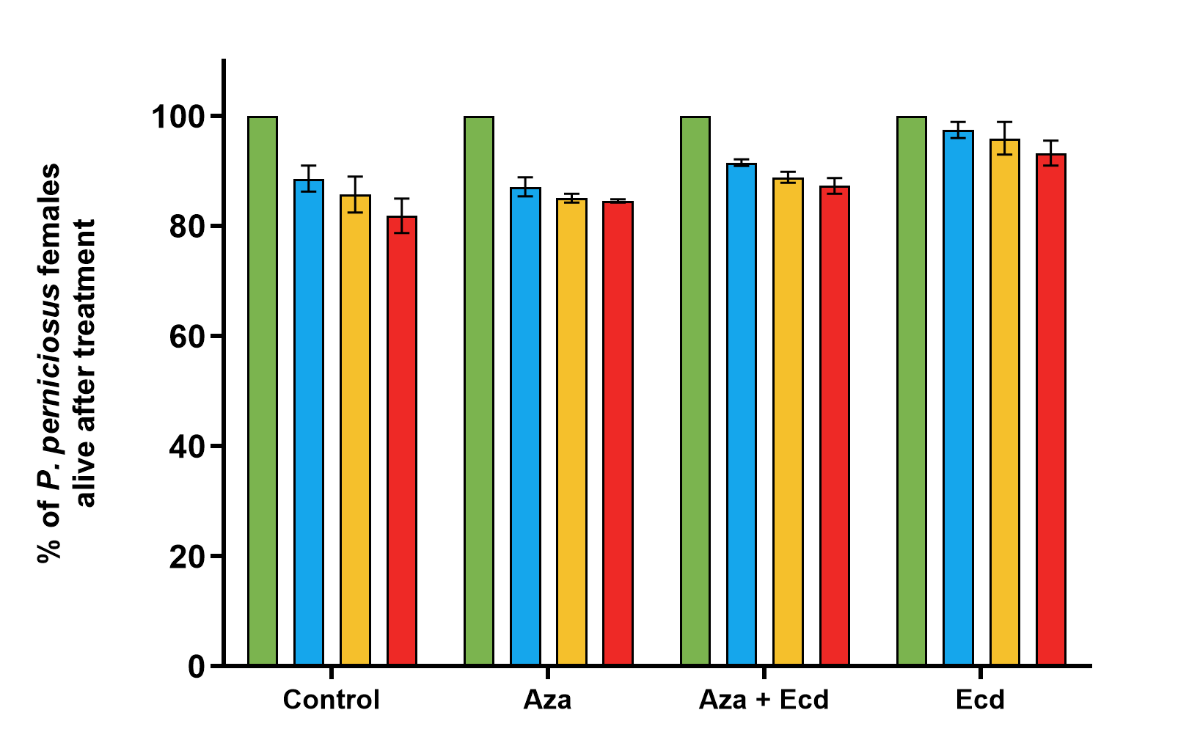


**Additional file 2: – Effects of azadirachtin and ecdysone treatment on the mortality of *P. perniciosus* females. Females were previously fed on sucrose solution containing: 0.9 % NaCl (control, green column), azadirachtin (1 μg/mg, blue column), azadirachtin (1 μg/mg) combined with ecdysone (2 μg/mg, yellow column), and ecdysone alone (2 μg/mg, red column). Mortality was evaluated 3 days after feeding.**
