# Supplementary material for: Azadirachtin disrupts ecdysone signaling and alters sand fly immunity
Source: Parasit Vectors. 2024 Dec 20;17:526. doi: 10.1186/s13071-024-06589-8 (PMC11662615; doi:10.1186/s13071-024-06589-8)
Supplement: Supplementary file 2 — Supplementary file 1. [file 13071_2024_6589_MOESM2_ESM.docx]

**DEFENSINS**

**Defensin 1**

AAAATAACAGGGGGAGTTCAGGTGGATTAACAAATCGTTGTAGACTTGCAGTGAATCCTAAACAAAGTCT

CTCTCAGTGAAAATCGAAGATGAATACCAAATTGTGCATTACTTTTGCTGTCCTTATCGCTATTGTGGTA

GCTGATCCTGTAGAGAAGAGGACTGAAAAAACTGAAAGTTTCTCAGGACTCTCTTCGGAGGAAGGTCCCG

AATCAGTGATTCAGCCTCGTGTGAGTTGTGACGTCCTGGATCCAACAGGATGGGGAAACGCAATTTGTGC

CGGACACTGCATTGTTCTCGGATACAAGGGCGGTTATTGTGACTCTAAAAAAATATGCAGGTGTCGTCGT

TAAGTTCAAAGAAGATTACCTAAGGAGAGAATTTAAAAAAAAAATGTAAAAATCCTTGAGAAATAAATAA

AATGTTGCAAATTTAGCTGAAATCTGAAATAACTTTCGCAATAACTTTTTGCGATTTGCGTCACTAACAC

GTGGATTTATTAGAAAAATTCTAAAAATTTACTTGTTGGATGAAAAGAATTCACGAAGAATTGACTAAGC

ATTTTATTATTGCATTGTGTACACGTACAG

**Defensin 2**

CAGAACATCTCGTTCAAAAAGATCTCAAAAGGTTTAAATTAAAACTTGTTAAAATGCGTGTTCTTTTGAT

CACCTTTGTTGTTGTGGCTGTTGTTGGAGTCGTCAGTGCTTATCCCTCGAGTTTTGTGGGAGACTTTGAG

GATTTGAATGATCAAGTTGCTGATGAAGTAGGATTCCCGGAAGCCCTATTTGGTCAGCCTGAGATTCATT

CTCGCCAGAAGAGAGCAACTTGTGATCTTTTGAGCGCTTTCGGGATCGGACATGCCGCTTGTGCTGCTCA

TTGTATTGGACATGGTTATAGAGGAGGATGGTGCGACTCTAGGGCTGTGTGCAATTGCAGGAAATAGAGT

TTTAACAACATTTTGCTGAATCGGGTCCATCAGAACTTGATGGAGACTTTTGTATTTTTTATTATATAAT

TAAATT

**ATTACIN**

GCTCGTGGTACTCTGTTCAACAACAATGGGCATCGCGTTGATGGACATGGGAGTGTCTCTCGTCAATGGCATCCGACAGGACCCACAAGCATTGGAGGAGGATTGGATTACACCGGACCGAGGGGATCTGCTTCCGTGAATGCTCAGCATCAGCATCGTTTCGGCACGAGTCTGACTGCCGAAGGACGTGCTAATCTCTACAGAAGCCCCAA

**ECDYSONE RECEPTOR**

CCTCAGAGCAGTTCGGTCGTGTGACTAAAAATACAACCCATAGTGAAAAGGTGTTTCTCTCTCACATTTT

CTACACTGTTGTGTGAGCCAAATTTGATATTTGGACATTTAATTCCAGCACTTGGAATAAAGAATAATAA

AAAACACTCTCTTCACAATCTCGTCACATTATTTTCTATATACATATACTCTGTATAACATTTTCACCCA

AAACCCCCCTGTGTTGCGGATAAAAATGTGTCTTTCTGTGTTTCATAAGTGGCAAAATTAACCGTGAGAA

GGGAGTTTAATTTCATCAAAAGTGTCTAAGAAGTAAAATTTCGAGTGTTAAGTCATTATATACCGCATTA

TATTATAAAGAGAAAAACTTGGAAAAGTGTGGAGAAAAACACCGGGAAAACATATTCTCGGCCGAGGAGG

GTTTTTATTCGGCTAGAGACTATAGCATCGCGCATACACAATTGCAAAGCCACATAGAACATAAAAATAG

TGGTGAAATGTGTTGATACTGTGTTGGTGAATGAGGTTTGGTTGGATGATAAACTATCGATTGGCAATCA

ATTTTGTGCTCTATAGACTTTTGACTCTCAAAACCCAGTGGAACAACTCCTAGTGCGCTCCATTGACCAA

CAGTGTTCTATTTGTCGTCAAATCGCAATCACGTGCTGTAGCACTCAAAACGGACTCTATAGAGAAATTT

AAAGAAAAGAAAAAAAAACTTTATTGAGAGTTCCATTTAATTCCACGCCAGTGACATTTTAGTGGAAGTG

CAATATTTACAAGAATCGCGAAAAAAGAGAAGACAATTCACTTGAGAGTTATAATATTTATGTTTCCAAT

TGCGTAGAATAAGAGGTGAGAAATTAACTCTACTTGTAAGTCATTGTAATTGTATAGTGTGTAAAGTGTT

GTAAAGAAAAAAAAACTGTCCAAGAGACTTTGACTTTTTTCTGTTTCGTGTGTGAAAAAAGGAGGATCAT

ACTGTTGTGCAATCATATCAGTGTTGAAAACAAAAAGAAGTGAATCGACACTCGAGGAATTCACTGTGTA

AGCCAAGTTGAAAAAGAAAAAGTGTTGTGTCGGCTGAAAAATATATATATGGATAACATTTTATGTATGC

TAATGACATTAAATGAATTAATATTCAACTGAAGGATCAACAGGGGTGAAGCAGCATGTATTGGAAAAAT

TCAGAGAGTTCAGCAAATCCCTATTTGAAATTAGGTGATTACGTTGCAAGTGGAAAGAGAGAATTGGCAC

CAAGTGTTCTTTGCTAATTAACAGAAGAGGAAAAAAGGGGGCTAATTGCAAAGAAAAATTACCACATAAC

ACAGAGGTTGTGGGCCAAGAAGAAACGTCCATACTGCATCGTTTGTTGAGCATTTCTTTCTCATTTATTT

TTTGCCCAATTAACCCCTCCCATCTCCCCCCCTCGACAACCACCCCGTTGACACTGTGGAGTTCGCGAAG

GGGTTATAGTTGCTGGCAACTGAAGCGAGATTGAGGCAAAATGTACCGTGTAAATTTAGTCAATCAAGTA

CCACCCCCACCAGGTGCGCCCCAACAGCAGCAGCTCGTTGGGGTGCTGAGTCAGATGGATGCAGGTGGGG

GTGGTGGGGGTAGCAATGGGGTCATGGTGGGCGGTATGGGTGGCAGCAGCTCGGCCAGTATGGGTGAGGA

TGCCATTATGACAGCTGTCAAAGTAGAGCCAATACACGATACCGCCTCCACAAGTTCAACAACCAACGGT

CACGTGGTCTACACAACGAAACGACCCCGCATGGAAGGTGACGATTGGTTGTCATCGCCTAGTCCGGGTA

GTGTACCCAGTTCAGCACCACCTCTAAGTCCATCCCCAGGTTCCCAGAGTCATAATTACAATAACATCTC

TAATGGATATCCATCACCAATGTCGACGGGCAGCTACGATCCCTACAGTCCCAATGGCAAAATAGGTCGT

GAGGATTTATCACCGTCGAGTAGTCTCAATGGCTACAGTGCTGATGGCAGTGAAAGTAAGCCGAAGATTA

AGAAGGGACCGGCCACGAGGCAACAGGAGGAACTCTGTCTCGTGTGCGGCGATCGAGCTTCCGGCTATCA

CTACAATGCTCTCACCTGCGAAGGATGCAAAGGCTTCTTTCGGCGGAGTGTCACCAAAAATGCGGTGTAT

TGCTGCAAATTTGGGCACGAGTGCGAAATGGATATGTATATGAGGCGAAAGTGCCAGGAGTGTCGACTGA

AGAAGTGTCTGGCGGTGGGAATGAGGCCAGAGTGTGTTGTGCCTGAGACGCAGTGCGCCATCAAGAGGAA

GGAGAAGAAGGCACAGAAGGAGAAGGACAAGCAGCAGGTTCCACAGCCCGTGAGCACAACGGACACGTAC

AAGACGGAAATTCTGCCGCAACTCATGAAATGTGATCCACCACCCCATGCGGCGTGCGCCGTAAGGGCAC

TCCTACCCGAAAAGCTCTTGGCTGAGAATCGGGCACGAGGCATTCCATCGTTAACGACGAATCAGATGTC

AGTCATTTACAAATTGATATGGTACCAGGACGGCTATGAGCAACCATCCGAGGAGGATCTCAAGAGGATA

ATGCACAGTTCACCGACTGATGATGAAGATCAACATGACGTTCACTTCCGACACATTACAGAAATCACAA

TCCTCACAGTACAACTGATTGTGGAGTTTGCCAAAGGACTGCCTGCCTTCACGAAAATCCCCCAGGAGGA

CCAAATTACTCTACTCAAGGCGTGCTCGAGTGAGGTGATGATGCTGCGAATGGCCCGGAGGTACGATGCG

GCGTCTGACTCCATCCTGTTTGCCAATAATCGCTCCTACACGCGAGATTCGTACAGAATGGCGGGCATGG

CGGACACCATTGAGGATCTGTTGCACTTCTGTCGGCAGATGTACTCAATGACGGTGGACAATGTTGAGTA

TGCTCTGCTGACGGCTATTGTGATCTTCTCGGATCGACCGGGATTGGAGCAGGCGGAGCTGGTGGAGGCC

ATCCAGAGCTACTATATCGACACATTAAGGATTTACATTATGAATAGGCATGGTGGGGATGCTAAGTGTT

CGATTTTGTTTGCCAAACTCCTCTCCATACTCACGGAGTTACGGACGCTGGGCAATCAGAATTCCGAGAT

GTGCTTCTCGCTGAAGCTCAAGAATAGGAAATTGCCACGCTTCCTCGAAGAGATCTGGGATGTGCAGGAT

GTACCGCCGGGACTTCATCCTCCGCCACCGGTGCTGGCGAATGGTCAGACAACGGGTGGGACAATTGAGC

CGGCATAGTAAATGGGCTCCACTCCCGTTAAAATCCTCCCCACAGGCAGCCCCATCGGCTCTCACTCCCC

CAACCATTGAAAATCCAGAGAGAGAGAGACAGGATAAAAGAAAAAAATTATTTATTGTAAATAGAGAAAT

TGTGTAAATGTATTTTGAAGTCTAATGTTGTTGGCAGAGACAAAAGATCTTGCGACAGTTTTCAGATTGA

CTCTTTGGATTGAGAAAATGTGGGAGGGGGAGGGGGATAAAGCCGAAGGGGTTTTTGCCTCAAGTGTTGC

GATTAATGTGGATGAAAACTAAAAGAAAAAGAAAGAAGTTATGTTGAGTGGAGCCCATTTGTGGTGTTTA

AATTTATTTTCTCTTATTTTCTCTAATAGTCCTGTGAAAGTATTTGTTGTGCCAATTTGGGAGGGAATGA

GAAGGGGAGAAAAACAGCGGTTTTTCTTCACGAATTTTCCTCCCAAATTGCGTCCAGAGCATTTTGACAC

GTAAATAGTGAAAAAGTTTAAAAGATGAAAAAATCAAGAAAAATAAGAGGAAATGAGATCGGAGAGAAAA

AAGTGCTGGAAAGGCTCCTTATCGTTTGGCTGTGACTTGTTATGTAGGAAAGCTATATAATTCTTAAAAA

AACAAGAAGATGAAAGAAGAAAAAAGTAATTTTAGACAGAGAGAATGACCAGAAAAAAAACTATTAGGAT

TCTAACTTGTAAAGTAAATAATGAGGATAATGAAAAGAGAGAAAAACGATATTTAATCATTTAAAGAAAG

TATAAAATTTTAAGGTAATTAACTTAGTAATTCAACTGCAGATTTTATCTTGTAAAATCATCTTGGAAAG

AGAGAAAATGTGTTTTAGGATAATGTTTTTTATTATTATTTTGTCATTTTAATTTTTGTATACGTCTTTC

TTACGATTTTATCTTTAAGAGAGAGATAAAGAAGATTTTTAAAAACAAAATAAATAAGAGGCAAAAATTG

GGAGGATGCGAGAGTGGCCAGCGACATAAAGGATAAATATTGACGAAAAAATTGCATCAATTTTTATCAA

CATTATTCTTTTGCGCATCAGTCTGAAATGAGTTGCAGCTGATAGGTAAATTACACTAAATAGAAAAAAA

GATGATGAAAAATCACAGCAAAAACAATAGAAGTGACTAAAACGGTTGAATGTCTCATCACATTGAAGAA

AAAACAGCAACAACCTTTTACAAAGAAAATACAAGTAAAAAAAAGAGTACGAAGAATTTGGAACGAGGTG

CCTTTTGACAAAAAATATTATTAAACTACAAAAAAAAGAGTGGAAAAATGCTAAAAAGGGGGCGGAAGGG

AAATTTGCTGAATGGCAACAGAGATTATTTTCATGGAAAAGAATCACAAGTTGATGAAGAGATAAAGGAA

AAAAATTCTATTGACTGAAGGGATTGGTGCAAGAAAAGTATAAAAAAAATTCAATAAATTATTTCAGACA

CTATTTAAGAAGCTCTGTATAAAAAGTCCCAAAAGAGGAAGTAAAAAAAGAAACCCAGAGAAGATAATTA

AGGGAAAAAAATAGTGTAGAGAAAGTAAAAAAGGCTGATTGTTTGATATTTTATAGGAGAAAAAAAAACA

TGGAAATTTTGATAATGGGACAAGAACAAGATAAACTTTTAACACAGATGAAGATTTCGAGGAAAAACAA

ATGAAGCAAGAAAAGATCAGCAGAGACGAAATTTCGGTTGGGTTGTGTTTTATTGCAAATATTTTGTGGT

TTTATTTTATAATTTTATTTTTCCCACTTTCTTTCTTTCATTTGATTGAATCGTGACTCTAGCTGAATTG

TAAAAAAAAACTTAAAAGAAAGAATATCTTTTATTAATTTCCTTTTAGCGGGATTTAAAATATAACCGTT

GCGAGAGAATAAAAGTTGAAACACTTTGGAACTGAATTGAGAAAACCGTCCGTTTTAGGAGAAATTTTCT

TTTTATTTTATTGTATACACGTTTATTTTATCTTGATCATTCTTTTTTTTTTTAAATAATTTTGTGGCAA

TCTTGGAGATGGTATAATTTTCACCCGGAACACGTCAATAAAGGAAAGGCTTAATGAATTGCGTTTGTGG

AAAAGAGCTGAGAAACGTCGCAACGTCTGAAATTTTCACCTCTTAAGTCTAAAAAAGAAAAAAACAAATC

GAGAGTTAAGAAAAAGAATCATTAAATAGACATACGTAAATTATATTAGAGAGAGAGAGAAAAATGAAAA

CAAAAGTGTTAAAAGTTTCAAAAATATATAGAAGAAAAAATCATATATTTAAATATCCTATTGATTACCG

GCAGACTAAATACCTATTGTTGAATGGAAAAGTCATTGCAAATATCTTGCGAAACTGAAATGGATCATCC

AAAATGCAAAGTGTTTAAAAAGAAATTTAACTAAAAAGAAAACAAGTATAAAATGAACAAGATGGGGGAG

ACAAAAACAAGTTTGACTAAATAGAAAATGGGCATAATAAAATGTACATAAATTAATTAAAAAGATGAAA

TTCAGATGAGACAGAGACATTGCGTAAAAATATCTTTAAAGAAAAAAAAAAGAGAATCACAAGAATTAAT

TAACACAAAAATATGAATAATTATGTATAACTTAGGAGATATATAATTAACATATTCTATAACCGTTCAC

AGAAATCAACATCTAAAATATTTATATAGTCTTTCCATTAAAAAAAGCGTTGAAATACGGATAAAGTAAA

ATATGGAAGCAATTAGTAAGTTGTGTAGATCACAGTGGAACTCTGCTAATGTAAAATCTATCAAAAAAAA

CTTTTGATGTGTAAAAAATAATCCCATTTTAAAAGTGGAATTCAGTTGCAATTTTGATGGTGAAGATGTG

AAAATGTCCAAAGGATTTCATTTTTCTTTATTTTTCTTTTTTCAAATTATATTGGAATCAGTAAAATAGC

CTGCAAAGAGTAGATTTTAGATCTCAAATAGTTGAACGACAAAATGCCACCATGCCTTAAGGGGTACCTG

TTTGCAAATGATAAACCATTGTTGACAAAGAAAAAAGAAGAAAATACGACAAACACAACAAAACCAGACA

TGAAACACGCTCAGATTAGAAGCAACATTATAAAAGCCATCAAGTACCTCACTCTCTTGATAAAGAAACA

TAAAAACAAAAAAAAATAATATTCAAACAATATAACTAAAATAGCAGAATAGCCATTCATCTTGGAAATA

CGATTCAGAACAATCAATGAACGGAATTTTTTATTTCAACAAAGATTTCTTGGGGTGAATTTTCAAACAA

AGTTTCTGAAAATTCAGCAAATTATTGAAATGACTGTCAAAATGACAGAAAATTTAGTAAATCTTCATAA

TTTGACTGTCAAACTGGTTGAAAAGTCAGAGAACTTTTGAATTTAACTGTAAAATGCGCAAAATGAGCTC

AAAATGCAGTAATTGTTCAAATTTGACTGTCAAACTGTCTAAAAAGTTAGCGAACATTTAAATTTAACTG

TCAGATTGACTGAAAATACAGTAACTGTTCAAATCTGACTGATATAAACTAGCTGAAAAGTCAGCGAATA

TTTAAATTCAACTGTAAAATTGGCTGAAAACGC

**SERPENT**

ATTTAGTACATAATAGTATTTTTTTTAATTGAGTGTTTATGTGAAGTGAATGAGACGTGAATATGTATGT
GAGGAGAGCTCCCTTAAGGAATTGTATTTTTGTCCATGCAAGTAATATTTTTCACGTTATCACTCGAACA
AATTGAGTCACTTCCTGTTGAAAGCAATGGGTGGTCACTCGAAACTTCCTCAAACTATATATCCAAACGA
ATTTTGCCCCAACATTGAGACTCTTGTTGGATTCTCAGGAGGAGCATCTCAAGTTTTTTCTCATTCCCGC
CTTGAGACATTCCATTGCAAAATTTCGACGACGAATATAGAGAGAGAAAAAAGTACAATTTTAATGGGAA
GCATGACTGAGTTGTGTATATATTTTGGAAATGTACTGCATATCTTGAGATTAAGCGTCTGAATATCATT
TGCTGTGATTTAAACGAAACAATCATTATTAAAGAAAAATTACATTAAATGATGAATTCAAGTGGGTCTC
GAAGCTGATTTGTTCACGGAAGGTCGTGAATGTGTAAACTGCGGAGCAATTTCGACACCTCTTTGGCGAA
GGGATGGGACTGGTCATTATCTGTGTAACGCTTGCGGTCTTTACCACAAGATGAACGGCATGAATCGACC
GTTGGTGAAGCAGCCAAGAAGATTGAGCGCATCTCGTCGAGTGGGTCTCTGCTGCACGAATTGTCGAACA
ACGCAAACATCTCTTTGGAGACGAAATGCTCTCGGCGAACCAGTGTGTAACGCCTGCGGCTTATACTACA
AGCTACACAACATTAATCGCCCTCAGACGATGAAGAAAGACACCATTCAGACTCGCAAAAGGAAGCCAAA
AGGGAGTAAAAACTCATCTGATTCCAGTGGATCGTCTTCCAAGCATCAATCCATATCTGACGCATCAAAA
GAATTGCGTGCGCTGAGTGCCATTCATCACACGACGCAATTAAACAGTGGAAATTCCCATCAAAGTGGAA
ATACAGCGTTGACCAGTTCGACTTCGCCTCAGCAGAATCTCTCGCCCAATAACCATCAAAATCTCTCACC
ACTCCCCTATTCGCCCCAGGAAGCGTCTCCGGGCGTCGTGACGGCGGCCAATATGAGCCTGGGTGCGCCT
AATGCTAGCAAGTTCTTGCAGAAATCCCTATACGGACAGATCGCCACCAGCGGCGCAAATTCTGGCAATT
TGTACCAGAATCCCGGACAGGGATACAGTTCATCGGCTTCCAACATCTATTATGATATTATCAGCAACTC
CATGTCTGCGAATCACAGCAAAATCGACAATCAACACATCTCGAGGAGTCCATCTGTGGAGGATGAGCAC
GATTGCCAACATGACATGATAGCGCCCCATAAAAACTATTCCGTGAAAATTGAGTCGGAATAAGAGGGGG
GCGCGCAAAGGGGTGGAAAAATCACTGTTATTTGCTCTAATTACGAATTTGATAAGAAATTGTATATATC
CATTTAGCAAATAACTTCTAAGCTTATTTTCTAATTTCATTTATATCGATGATATCCATTATAATTAAAA
ACCTGTGTTATTTTTT

**Eip74EF**

ACACGATCAGTCAGTCGATGACTTTTGCTTGGTTTGAACGCGGTCGCGATTGTGTTCTTCGCAAATGCAC
GTGAGCGCGCATTTTTCCGTGGTTTTGTTTTTGCTTTGAGAGCATCCAATTTGTAGTGAAAGAAAAACTT
CCGTGTCACGGGGGTGGTTTGAGTTTGAAAATTGTCCGTTACTTTTGGCGCCAAAACTTTGAATTGTGTT
TTTTTGATAAAAAGAAAAGCCTTGTGACTGTGCCTTAGTGTCTTCCGTGGGATTTTTTCCACACACACAC
CTTTGGGATTTAATCAACATCCTGACACACTTTTGGTCAGGACTTGGATTTAAGTGTTTTTTTGAGGTTA
AGTGCTCAAAGTGTTTTTCAAACGGTTTTTTTTGGCAAAGAAAGCGCCATTGCGCTTCTTGCAATTGAAA
GTGAATAAAAACAAAGAGGAAAAACTTGATAGAAGAGAAGAAAAAAGTGAGAAATAAAAATTAGAGTGAT
TTTTTTGCGTTATTGATATACAGAGAGTTAAGTGATAGCGTGATATTGCCCATAGAGCAATTTTTTGTTA
TCTCATTCTAACCACCTTCACTTCGGTCTCTCTCAAACCATTACTCACTTCGTTACAAGGACGAACATGT
GTATTTAATATGTTCCAATCTCGACTTTATACTTCCTTGTAGTATTTTTTTTTAAATAAATTTATTTCTT
CATCAATTACAAACCGAAACGTGTTCAAGGTTCCAAAATAAAACACTCCACAACATCCCAATACTTTTAA
AAGTCTGCGGAACTTTAAAGAAAAAAAAATTAATAAAAAAAATATAGTGACGAGTGCCTTTAATACTCCT
TTAATTCTCTGAGTTTTATTAAGTGTATCGAAGAGAAATTTTTATAACTTTTAATAGCTTAGGCTATTAG
TGACAAGTGTGTGTGTGACGGTTTGAAGATTTTATTTGGTTAAACCAGAAAAGATATAGTGAAAAAAGTG
CAAGATTGGATTAACACATTGTATGGGATTATCTCAATTGATTTACAATCCATTTGGATTAACCATCATC
TTCCCAGTCATCCCGACAAACAGTGATTCCACCTGAAGAGAGTTCATCATGCGATTGTCTGACGAATGAG
ATTTCCAAAAAAAAATTAGTGCCTCTTCAAGGATCAGTTTGAGCTTTTTCTTTCCAAATCCTGAAATGCC
AAGAGGACGGAAAAGCTCTCTCCCAAATGACGATCTTCGTCGATTAACACAGCTTACCACCGAATAAATG
AGCCACAGAGCTAACAAAAATGCCAACAATTCACCAACAAGTCGTGAAGAGTTCTTCGATACGTGATAAA
TTTCTGGATAAATTCCCTAAGCATAGGAAAATAATAAGAAGGATGTGAAACATTAGTGCCTTGGAAAGTC
AATAACTATTTTTTTCTCATTGTTAAGTGTTCTAAATTTTTTTTACAAACGAAAGAAAAGAAAAACAAAA
ACTGTGATTCTCAAAATTTTCTACAAGCAAAGAAAAAAACGTTATTTTAAAAAACATTTGTGAAGAGAAA
ACCGTTCAAAGTGATTCCAAAGTGCTATAAATGTGTCCTAGGAAACAACACTGCCTTATCTAATACATCC
ACCATCTCGAGAGCTAGTGAAACTGTTCAACAGCAAACAATATCGCGTGTTTGAAAAATAACGAAGACTG
ATTTACCGCCTCTTGACTTTTTTGCAAAATCTTTTTTTCCCGGTTATAAACTGATATAAAACGGAGTGTG
TAAGAGAAAATTTCCTTATAAAAAAAATTGATTATCGAGCGCTGTATTGAAACGCTACAGGGACGAGAGA
ATAGAGGGCAGTGCTGCTGTGGCAATGCCATTTATCGAAGACGAGCTCCTCTGGTGTCCAGATAACGATG
GGAGAATGGTGGATATCTCAGCTTGTCTTCAGGACGCCGTAACGGCGAACACGACGAACCAGAATCAGGA
GGCTGTCGGTGGTTCATGTGATTTGAGCTCACTGGATCCTCTTTGCAATGATTCCGATGAGATTCTCCGC
CAATTAGCCGAGAATCCCTTTGAGTTGGAATCCTTTTTCTCTGACTTCTCAGCGGTGGAAGTGAAACAGG
AGGAGAACAACAATGACCTGCCCGTGGATCCAAACGACAGTCCCACGTATCTGACCAACTGCCAAAGCGC
CTCGGCGCCCAATTCGCTGCCTCTGCAGACCGTTAGCACGACGTCCGTGAGCGCCGTGTCGCAAGCGGAC
TCGTCACAGAGTCACTCGCAGCAGCACCAGTCGATCCTGGCGCTTGCCAACGCCATCTCGGCGAGCTCCT
TCGGGTCTCACCAAACACAGAACGGACGCAATCAGGCGACCGTCAACAGATACTCCATTGCGGCCAATCC
GCTGCTCGCGGAGAAACTCATGGCGCCAAATCTCAATGACATGGACAGCAGCCTGGCCATCGGCAACCGT
GCAGGTCGACCGCCGGACGTTAAAGTGAACACAGAAATTCCCATCTTGCCACAATCACCGAGTCCGCCGC
CACAGCAGAGGATCTCAGCATCGATACCCGCGGCAAGTGCAACCAATGGCCACCACCAGGGACCACCGCC
CCATCGGCAACTCCTCCACGGACTGCTGAGCGGAGCGCCGATCCACACAGCGCCATACCATAGGAATTAC
AGCACGTCAAGCACAGGTTCACTGCCACCGAGTCCGGCGGATAGTGGAGTCTCTGACGTGGATTCGTCCA
GTTCAGGTGGACAGCCGGCGTGCAGTGAAGAGCTGAAGGCGCGCCTCGGGATGCCACCGCACTGCCCGCC
TCAGGGCCACATGGCGCCCGGCACTTTCCTGCATCCCAACTTCTACCACAACTCACCGCCACAATTACGT
AATATATGGAATAATCGCAATGTATCACTGCCAGACAGTTACTACCTGCATTCGATGAACGGTAGTTATC
CGCCATCGCACTTTCCGACCCCGTCACCCGCCCGCGTGGGCCCCCACCAGGCGCTCCATCCGCAGAGCGT
GATCCAGGCGGCGACGTCGAGTGTCGGCGACGACATATCGTACATGCTGGAGTTGGGCTTCCAGCAGCGC
AAACTCAAGAAGCCCAAGAAGCCCCGAATTGAGATGGGCGTCAAGAGGAAGAGCCGAGAGGGATCCACCA
CGTACCTATGGGAGTTCCTGCTCAAACTGTTGCAGGACCGTGAATACTGCCCGCGCTACATCAAGTGGAC
GAACCGTGAGAAGGGAGTGTTCAAACTGGTGGACTCTAAGGCTGTGTCGAGACTATGGGGTCTGCACAAG
AACAAGCCAGACATGAACTACGAAACCATGGGTAGGGCCCTACGCTACTACTACCAGCGAGGTATTCTGG
CCAAGGTGGACGGTCAGAGACTCGTTTACCAGTTCGTCGACGTCCCGAAGGATATTGTTGAGATTGACTG
CTCCGGTGCGTAGACGCTTCTCTCTATCGCTTTCCCCCACTCTTTGTATCTTCCCCCACTCCCCGGACTT
TGCGAACAAAACAAGCGCGCGCGCCCCCTACTTATTTATTTTCCAAAACTCAACGCATAATCAAAGATAT
TCCTTCCACCATCTCACCAATGAAAGGAAGATTAAAAGAAAGAAAAAAACAAATATATAGAAAGAGAGAG
TTTAAAAAATATAAAACACAGAGAATAATAGAAAAAAGTCTAATATCATTAATAGAGGTTATATATTATT
AAAAAAATAAAATTATTCCTCTCTTTGACGGGATGGAAAAAAATACTTGATGTTTTTTATGTTACCTACG
TAAAAGTTATTACCTTAATTTAAAGATAAATCTTTTGAAAGAAAAAAACTAAATATTAGGTTAACTTAGG
ATAGGTGGTGGTGATGAAAAACAATAAATTATCAATATCAAGTTTTTTCGAATAAATGAATTTGCCTAAA
TTCGGCATCGCTCTAAATTCGCTGCCAAATTGTTGAAAAAAA

**Eip75B**

CTGCTCGTCGTGTCATTTGCAAATGAACCGTTGAAGCGCGCACACCACACGAGAGCTTTTTCAAACAGAG
CAATCGCGCGCCCCAAAAGTGATATATACATATTTTAGTGAAGAAGTGATTCCTGCAATGCAAGTTGTGT
GATAGGCAACAAAACCAAGTGCAATTGTGGCCCAGTGACCCTAGCCTGTGTGGGGGTGTTAAAAAGTGAT
AAGTTACCAAGAAGGGGAATGGGCTGTGCAATGCAGGAGATGGCGCCCCAGACGAATGAGGACGAGCGGA
GGGACAGTCTAAAGGACAGTCACAGTGTTCTCGTGCAGATGCTGGAGGCTGCCCCATTGGGGGCGCCTGT
GTCACCCCGACACCATCACTACAGGAAGCGACTAAAGGGGGCTGTTTTGGGCACGTCTGAGTGTCCGTGG
AAGAAGAGCAGGATTGGATGGAGACGAGAGGAACAACCAGAACCCGAACGACGGGATTCCTCGGACAGCT
CCGAAGGGGGTCCCAGTGATTCGGGATGCGACAGTGATTGTCCGGAGAGTCACAACATCACGGAGCTCTG
CAAGAAGTTCGACGAGAATCTGTCGGAAGATGGCTTCTTTCGGCGATCAATTCAGCAAAAGATCCAGTAT
CGGCCATGCACGAAAAATCAACAGTGCAGCATCCTTCGGATCAATCGCAACAGATGTCAGTATTGTCGCC
TCAAGAAGTGCATAGCAGTGGGAATGAGCAGAGATGCCGTGAGGTTCGGGCGGGTACCGAAGCGCGAAAA
AGCGCGCATCCTTGCGGCCATGCAACAGAGCACACAGAATAGGGGCCAGCAGAGGGCGCTCGCCACTGAA
CTGGATGATCAGCCACGACTCCTGGCCGCCGTTCTGCGCGCCCACATTGACACATGCGAGTTCACCAGGG
ACAAGGTGGCGACGATGCGTCAGAGGGCTAGGGAGTGCCCGTCCTACTCCATGCCAACGCTGGCCTGTCC
GCTCAATCCGGCCCCTGAGCTTCAGTCTGAGCAGGAGTTCTCGCAGCGTTTCGCCCACGTGATTCGTGGT
GTGATTGACTTTGCGGGTATGATTCCTGGCTTTCAGTTGCTCACGCAGGACGATAAGTTTACGCTGCTCA
AGGCGGGACTCTTTGATGCGTTGTTTGTGCGTCTCATCTGTATGTTTGACAGCTCCATCAATAGCATCAT
ATGTCTAAATGGTCAAGTGATGCGACGCGATGCCATTCAAAATGGCGCCAATGCACGCTTCCTTGTGGAT
AGTACGTTCAATTTTGCCGAGCGCATGAACTCGATGCAACTGACAGATGCTGAAATTGGACTATTCTGTG
CTATTGTGCTGATAACACCAGACAGGCCGGGATTGCGCAATGTTGAGCTGATCGAGAAGATGTATACGCG
ACTTAAGGGTTGCCTACAGACGGTCATCACGCAGAAGCGGCCCGATCAGCCCGAATTCATGGCGGAGTTG
CTGAAAACGTTGCCAGATCTGAGGACACTGAGTACGTTGCATACGGAGAAGTTGGTGGTGTTCAGGACGG
AGCACAAAGAGCTGCTGCGCCAGCAGATGTGGTCGATGGAGGAGGAGGCGAGCAAGAGTCCTGGCTCCAG
TTGGGATAATAGCGATGATGTGGCCAAGAGTCCAATGGGCTCAGTCTCCAGTACAGAGTCTGGAGATACA
AATTCAGAATTCAGCCAGAGTCTGTCCACCACGGCGCCCCTATTGGCCGCCACCCTGTCCGGTTGTCCGA
TGCGCTATCGCGCCAATTCCGGCTCCTCCGAGGATGATCTCGTGGGCACAGCCCACCTACAACAGAATGG
CCTCACGATCACACCGGTGGTGAGGTCGACGGGACATATTCGCTACAGGAAGCTGGACAGTCCCACGGAC
TCAGGTATTGAGTCAGGCAATGAGAAGGCGGATCACAAGGCGAGCAGCGGTTCCAGCTGCTCATCACCCA
GATCGTCCGTCGAAGATGCCGCTGAGGAGAAGAAGTACATGCCAGTGGAAGATATGCCCGTCCTCAAGAG
GGTTCTCCAGGCACCGCCGCTGTATGACACAAATTCGCTCATGGATGAGGCCTACAAGCCCCATAAGAAA
TTCAGGGCGATGAGACAGAAGGACGCTGAAGCGTCAGAGGCTGAACCTGTTCAGCAGCAGCAGCAACAAT
CCCAATTGCAGATGCATTTAACGCGACCCGCCGCCCACCAGTCTTCCCTCTCGAGTACGCATTCCGTGCT
GGCCAAGTCACTGATGGAGGAGCCCCGCATGACACCGGAACAGATGAAGCGCACCGACATCATTCACAAT
TACATAAAGCGCGAATCAGCGGCTGAACAAGCGGCCTATCGTAGTCCCCATCATCCGGGTGGTTTGCTGG
TGTGCAGCGCGACGCAGGCGGGATGTCCCTATCCCGCCTCCCGGTGGCCGGTGATAACAACAGCGCGCCA
GCAGACACCGTCACCCAGTGATCCCCACCACTACTTCCAGTCGCCCCACTCCACGTCCACATCGCCACCA
GGTCCGTCGCCGTCGTCCAGCTCATCGTCGGCCTCGCCGAGACTCATTGAGCTGCAGGTGGACATTGCGG
ATCCGCAGCAGCCGCTCAATCTCTCCAAGAAGTCACCCACGCCACCCCCACGTGCGCCTCTCATCGCTCA
GGCGGTAAGTGGCCCCAGTGGCGCCCCCAAACTCCTCCTCGAGGCATAAGTCGAGCTCCCGCCCCCAGTG
TCGCTAAGATCTCGTGTCCTCCATCATGAAGACGCCATTTTTGTTTAATTTTTCGAAAACATTATTTTTT
TTTCGAACAAGACTTTCGATGTGTGGGTTTTTAATGTGCAATATAAAAGGAAATTTCTTTCAAGAGAGAG
AGAGAGAGAAAAGACACCATCGAAATTTGTTTTTCTTCCATTTTGGTGCTTTCTGTGCCCCCATTTTCGA
AAGTCACAGAAGCCACACATTTTGTTTTGACCAAAAAACTGACTAAAAGTCAATGAATTTTCGAACATAC
AATTTTCGAAAATGATAAATGGCGTCTCAAAGAGCACAACACTGTATAAAGAAAAAAAATTATTCACTTT
CGAGGGGTTTTTGGGGGATATTTTCTCCCATTTTCCGGGGGATTTTCTCCCCACCAAATGACACTCTCTA
AGGACCCCCAAAATCTCTCGGACAAGACACACACACAAAAAATGGTGGAGTATTGAAAACCAAATAATTT
ATTCGTATTTGTATGATAAGATGATTCTGAGGAAATGAGAAAAAAAAGTACGAGAGAGAGAGAGAGAGTA
AGAAAGAAAATTACAGAATTTCCCTTCTCAGAGAAAAATGCCAGAAATCAATTAGGGTGAAGAATGAGAG
AAAAAAACAGGATTTTACGTATGAAAAAATGTAGAAAAAAATCAGTGTATTTATGCAAAAACAGTTCGCC
ATCTCTCACTCTATTCTCCCGAAATTTCGGGGGGCTTATTTTGAGAGAAGTGCGCCTGAAGACATTTTAA
ACGTGGGTTATATACATTTTTTTCCGTAGAGGACTTGTAGTGTGAGAAATATAGATGGAAGATTTAAAGA
AACAAAAAAATATTTAATGAAAAAAATGCAAAAAAAATCAATTGTGTAAATGTGTTTAGAGTGCATGTTT
TCAATTATATCCCAATTTAGTGTTTAAGGAAAATATAAAAAAAAGTATATTTTAGGATTGGAGGTTATGA
GACAACCTCAAATTCAGGCTAGACGCTGAAGATTGAGGTTAGGAGCAGCGCAACATGTGTTGAAAATGAA
TATATCCATATTGCATTACAATTAAATGTGGGCGAAAGTGGAGAGAATTTTGAAGGAAAAAAAGAAAAAA
AAACATAATAGTAATTACTCTTAAATCTATGATAGCGTAAAAAAACATTTTAAATATCTGAAAAGAGATG
ATAAATCAATTTTCTCAAAC
